# Differences in Polyamine Content between Human Milk and Infant Formulas

**DOI:** 10.3390/foods10112866

**Published:** 2021-11-19

**Authors:** Nelly C. Muñoz-Esparza, Oriol Comas-Basté, M. Luz Latorre-Moratalla, M. Teresa Veciana-Nogués, M. Carmen Vidal-Carou

**Affiliations:** 1 Departament de Nutrició, Ciències de l’Alimentació i Gastronomia, Facultat de Farmàcia i Ciències de l’Alimentació, Campus de l’Alimentació de Torribera, Universitat de Barcelona, Av. Prat de la Riba 171, 08921 Santa Coloma de Gramenet, Spain; nelly.munoz@ub.edu (N.C.M.-E.); oriolcomas@ub.edu (O.C.-B.); mariluzlatorre@ub.edu (M.L.L.-M.); veciana@ub.edu (M.T.V.-N.); 2 Institut de Recerca en Nutrició i Seguretat Alimentària (INSA·UB), Universitat de Barcelona, Av. Prat de la Riba 171, 08921 Santa Coloma de Gramenet, Spain; 3 Xarxa d’Innovació Alimentària (XIA), C/Baldiri Reixac 4, 08028 Barcelona, Spain

**Keywords:** polyamines, putrescine, spermidine, spermine, human milk, breastfeeding, infant formulas

## Abstract

Human milk is the gold standard for nutrition during the first months of life, but when breastfeeding is not possible, it may be replaced by infant formulas, either partially or totally. Polyamines, which play an important role in intestinal maturation and the development of the immune system, are found both in human milk and infant formulas, the first exogenous source of these compounds for the newborn. The aim of this study was to evaluate the occurrence and evolution of polyamines in human milk during the first semester of lactation and to compare the polyamine content with that of infant formulas. In total, 30 samples of human milk provided by six mothers during the first five months of lactation as well as 15 different types of infant formulas were analyzed using UHPLC-FL. Polyamines were detected in all human milk samples but with great variation among mothers. Spermidine and spermine levels tended to decrease during the lactation period, while putrescine remained practically unchanged. Considerable differences were observed in the polyamine contents and profiles between human milk and infant formulas, with concentrations being up to 30 times lower in the latter. The predominant polyamines in human milk were spermidine and spermine, and putrescine in infant formulas.

## 1. Introduction

Human milk is the gold standard for human nutrition for at least the first six months of life since it satisfies all the requirements for infants to achieve optimal growth and development [1,2,3]. In addition to nutrients, this complex and highly variable biofluid contains nucleotides, hormones, growth factors, immunoglobulins, oligosaccharides, cytokines, and bacteria, which participate in the development of the immune system and provide protection against infectious diseases [2,4,5]. Additionally, human milk is relatively rich in polyamines, including putrescine, spermidine, and spermine, which are synthesized in the mammary gland during pregnancy and lactation and are reported to play a role in the hormonal regulation of lactogenic processes [6].

The few studies in the literature focusing on polyamines in human milk report highly variable concentrations [6,7,8,9,10,11,12,13], which may be determined by the mother–child dyad (i.e., the ethnic origin, age, nutritional status, and dietary patterns of the mother and the type of birth) [6,11,12,14]. The lactation phase and factors related to the breastfeeding process itself (foremilk vs. hindmilk and the time of the day), as well as infection in the mammary gland, are also influential [13,14,15,16].

When breastfeeding is not possible during the first year of life, human milk can be replaced partially or totally by infant formulas [17], whose compositional standards are established by the Nutrition Committee of the European Society of Pediatric Gastroen-terology, Hepatology and Nutrition (ESPGHAN) and the American Academy of Pedi-atrics (AAP). The aim is to achieve formulas that match human milk as closely as possible and cover the nutritional requirements of the infant for optimal growth [18,19]. Infant formulas are classified into two groups: those that are based on cow’s milk, including first formulas (those that satisfy the infant’s nutritional requirements during the first semester of life; EU 609/2013) and follow-on formulas (aimed at infants of about 4–6 months of age who have commenced complementary feeding; EU 609/2013), and formulas for special medical use (aimed at infants with digestion, absorption, or intolerance problems, or those who do not consume animal products for religious or other reasons) [18,20,21]. The latter group includes formulas for premature infants and formulas modified in carbohydrates, proteins, and/or fats; examples include lactose-free, soy-based, rice-based, hydrolyzed protein, and elemental formulas.

The occurrence of polyamines in infant formulas has been reported, although data are scarce and, in some cases, outdated. Moreover, most of the studies are focused on first and follow-on infant formulas [7,8,9,12,22,23], with only two studies analyzing preterm formulas [8,12] and one study analyzing soy-based formulas [7]. Due to recent changes in the formulation of these products, the available data on polyamine content may not reflect the current reality.

Polyamines participate in several biological processes, mainly cell growth and differentiation and protein synthesis [24,25]. Their role in the first years of life, in both the neonatal and infant stages, is important, as they promote the maturation of the gastrointestinal tract and help to maintain the integrity of the intestinal mucosa [26,27,28]. In this way, these compounds reduce intestinal mucosal permeability and the passage of antigenic macromolecules from the lumen to the blood circulation, thus reducing the risk of allergy in the infant [7,29,30]. Additionally, polyamines are involved in the development of the immune system and modulate the inflammatory response [28,29,31].

Due to the importance of polyamines in the first stages of life, the aim of this study was to evaluate the occurrence, profile, and evolution of polyamines in human milk during the first five months of breastfeeding. Moreover, the polyamine contents of different types of infant formulas retailed in Spain were determined and compared to that of human milk.

## 2. Materials and Methods

### 2.1. Samples

#### 2.1.1. Human Milk

In total, 30 samples of human milk provided by the Blood and Tissue Bank of Catalonia, Spain, were analyzed. These samples were taken from six mothers during the first five months of breastfeeding and correspond to a pool of the milk produced over a whole day. All mothers were from the same geographical region (Catalonia, Spain) and had full-term babies by natural birth. All samples were stored at −80 °C until the day of their analysis.

#### 2.1.2. Infant Formulas

In total, 15 different types of infant formulas available on the Spanish market as powdered products were selected. The formulas included were first, follow-on, preterm, and others designed for special use based on plant protein (rice and soy). For each kind of infant formula, three brands and two different batches were analyzed. The formulas were reconstituted according to the instructions on the label of each product on the same day as the analysis.

### 2.2. Polyamine Analysis

The polyamines were extracted from human milk and infant formulas as described by Muñoz-Esparza et al. [13]. Briefly, 1 mL of homogenized human milk or previously reconstituted infant formula was acidified with 70% perchloric acid and mixed for 20 min. Subsequently, samples were centrifuged (15,000 rpm, 4 °C, 15 min) and the supernatant was recovered and filtered through a 0.22 µm GHP filter (Waters Corp., Milford, MA, USA). Samples were stored at 4 °C until their analysis.

Putrescine, spermidine, and spermine were determined by ion-pair ultra-high-performance liquid chromatography coupled with fluorometric detection (UHPLC-FL), as described by Latorre-Moratalla et al. [32]. The chromatographic separation of polyamines was accomplished with an Acquity UPLC BEH C18 1.7 µm reverse phase column (2.1 mm × 50 mm) (Waters Corp., Milford, MA, USA), followed by online post-column derivatization with ortho-ophthaldehyde and fluorometric detection (ex: 310 nm and em: 445 nm). The quantification of polyamines in human milk and infant formula samples was carried out using the external standard method through a linear calibration curve of fluorometric response obtained from a range of standard solutions between 0.05 and 5 mg/L.

### 2.3. Statistical Analysis

The statistical analysis was performed with the IBM SPSS Statistics 25.0 statistical software package (IBM Corporation, Armonk, NY, USA). When analyzed by Shapiro–Wilk tests, the human milk samples did not follow a normal distribution, so the polyamine contents throughout the breastfeeding process were compared using the nonparametric Friedman test with Wilcoxon post hoc for paired samples. The one-way analysis of variance test, employing T3 de Dunnett, was used to compare the polyamine content among infant formulas, and the differences among batches were assessed with the Student T test. The level of significance was a *p* value ≤ 0.05.

## 3. Results and Discussion

### 3.1. Polyamines in Human Milk

**Figure 1:**
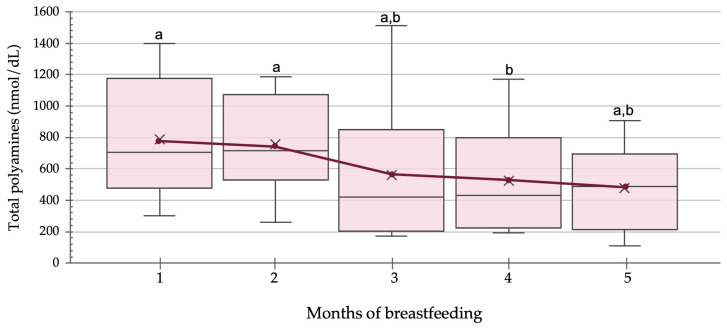
Distribution of total polyamine levels (nmol/dL) in human milk samples (*n* = 6) during the first five months of breastfeeding. The bottom and top of each box (interquartile range) are the 25th and 75th percentiles, respectively. The central line represents the median and X represents the mean. Lines extending vertically from the boxes (whiskers) indicate variability outside the interquartile range. The red line represents the evolution of mean polyamine content during the first semester of breastfeeding. The Friedman test with Wilcoxon post hoc were used to compare the total polyamine contents during the breastfeeding process. Different letters indicate statistically significant differences between breastfeeding months (1 vs. 4 months, *p* = 0.016 and 2 vs. 4 months, *p* = 0.016).

Spermine, spermidine, and putrescine were detected in all human milk samples collected from different Spanish nursing mothers. Figure 1 shows the distribution of the total polyamine levels found in human milk during the first five months of lactation. The total polyamine content varied greatly among mothers, with interquartile ranges oscillating from 544 nmol/dL to 699 nmol/dL. As depicted in Figure 1, the mean total polyamine content progressively decreased as lactation progressed, with levels at the beginning being 1.7-fold higher than those at 5 months (790 nmol/dL and 477 nmol/dL, respectively). However, according to the post hoc Wilcoxon test, the reduction in the total polyamine content was only statistically significant at month four in comparison with months one and two (*p* = 0.016). Other studies have also reported higher polyamine contents in human milk in the first months of breastfeeding, with values 1.3- to 3.5-fold higher, depending on the study, when compared to the subsequent months [7,8,12,13]. As polyamines are involved in cellular growth and differentiation, it has been hypothesized that their higher concentration at the beginning of lactation could be related to the rapid growth of the infant in this period [11,12].

**Figure 2:**
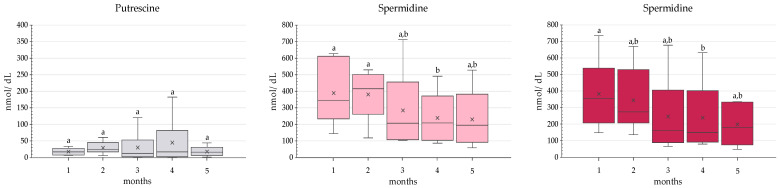
Polyamine levels (nmol/dL) in human milk (*n* = 6) during the first five months of breastfeeding. The Friedman test with Wilcoxon post-hoc were used to compare the total polyamine contents during this period. Different letters indicate statistically significant differences between breastfeeding months. For spermidine: 1 vs. 4 months, *p* = 0.016 and 2 vs. 4 months, *p* = 0.031. For spermine: 1 vs. 4 months *p* = 0.016.

Regarding the distribution profile of the three polyamines, putrescine was always the minor compound, while spermidine and spermine were detected in very similar proportions (a ratio of 1.1) (Figure 2). Putrescine has been extensively described as the minor polyamine in human milk, but consensus is lacking as to which is the most abundant, with reported spermidine/spermine ratios ranging from 0.9 to 2.5 [6,7,8,9,10,11,12,13]. Figure 2 also shows the evolution of each polyamine during the lactation period. Overall, spermidine and spermine levels showed a decreasing tendency, although their reduction only became statistically significant at month 4 (*p* < 0.05). On the contrary, putrescine levels remained practically unchanged during the first five months of breastfeeding.

On the other hand, abnormally high levels of putrescine were found in samples from one mother at three and four months of breastfeeding (121 nmol/dL and 183 nmol/dL, respectively), as well as an unusual occurrence of other biogenic amines, histamine (169.6 ± 5.7 nmol/dL), and cadaverine (2679.6 ± 78.9 nmol/dL). A possible explanation for the high presence of these amines could be that this mother suffered from mastitis, an inflammatory disease of the mammary gland that involves bacteria with potential aminogenic capacity. These findings are supported by the results of the work performed by Perez et al. [16], which showed higher concentrations of putrescine together with the presence of histamine, tyramine, and cadaverine in mastitis-infected milk in comparison with that of healthy mothers. It is worth highlighting that putrescine, in addition to a physiological origin, can be formed by microbial activity, as can histamine, tyramine, and cadaverine [33]. On the contrary, the sources of spermidine and spermine are generally physiological rather than bacterial [25,34]. Despite the abnormally high levels of putrescine in these samples, the overall polyamine profile was not affected due to the strong prevalence of spermine and spermidine.

The large individual variation in polyamine levels in human milk reported here is in accordance with the literature [6,12,14]. This high variability can be attributed mainly to the particular characteristics of each mother–child dyad, such as the ethnic origin, age, nutritional status, and dietary patterns of the mothers and the types of birth (whether natural delivery or cesarean section and term or preterm) [6,10,11,12,13]. Geographic location can also influence the polyamine content of human milk, as reported by Gómez-Gallego et al. [6], who found significantly higher polyamine levels in samples from mothers in Spain compared to Finland, South Africa, and China, which they attributed mainly to differences in dietary patterns [6]. Moreover, in the current study, the mean polyamine content in breast milk from Spanish mothers was notably higher than that recently found by Muñoz-Esparza et al. [13] in Mexican mothers, which could be explained by differences in genetics and/or diet associated with each geographic region. This supposition needs to be confirmed by further studies with a higher number of participants than are involved here.

Regarding the influence of diet, Atiya-Ali et al. [12] found a relationship between the maternal intake of dietary polyamines and their levels in preterm milk. Thus, the higher content of spermidine in human milk was significantly associated with a greater intake of vegetables and that of putrescine with fruits, especially oranges. These authors also observed an increase in polyamine contents in breast milk of obese mothers in response to a nutritional intervention with higher consumption of vegetables and fruits [10]. According to Muñoz-Esparza et al. [35], the major polyamine in most vegetables and fruits is spermidine, whereas putrescine predominates in citrus, which supports the findings of Atiya-Ali et al. [10,12]. Therefore, if the influence of diet on polyamine levels in human milk is confirmed, supplementing the maternal diet with polyamine-rich foods would be a simple and effective way of boosting the polyamine content in human milk. The best dietary sources of polyamines include mushrooms, soybeans, wheat germ, broad beans, green peppers, and citrus fruits [35].

The need for polyamines increases in periods marked by rapid cell growth, such as the first years of life or after surgery [12,36,37] and, therefore, probably also during fetal and infant growth and development. Accordingly, the diet of pregnant or lactating mothers could be adjusted to increase the supply of exogenous polyamines.

Another factor that could influence the polyamine contents in human milk is the time of sample collection, i.e., whether it was during the day or night and/or at the beginning or end of the feed [11,13,15]. In a cohort of 83 Mexican mothers, Muñoz-Esparza et al. [13] found significantly higher concentrations of all three polyamines in human milk obtained at the end of the feed (hindmilk) than at the beginning (foremilk). Unfortunately, in the current study, this comparison could not be made because the samples were obtained from a human milk bank and correspond to the total volume provided by each mother.

### 3.2. Polyamines in Infant Formulas

Spermidine was found in all analyzed infant formulas and putrescine in 86% of them. In contrast, spermine was only detected in two premature infant formulas. Table 1 summarizes the polyamine content of 15 commercial brands of infant formulas belonging to five different categories (first, follow-on, preterm, rice-based, and soy-based). In general, the levels of putrescine and spermidine were very similar among all the first, follow-on, and preterm formulas, with values always lower than 60 nmol/dL, except for one preterm formula, which contained spermidine levels of up to 230 nmol/dL (*p* < 0.05). The low content of polyamines in cow’s milk may account for the low values in these infant formulas [34,35]. No statistically significant differences were found among the batches of any of the analyzed brands of infant formulas made from cow’s milk.

To date, few studies have analyzed the polyamine content in infant formulas [7,8,9,12,22,23], only two of which have included preterm formulas [8,12]. Overall, the polyamine data for infant formulas in the literature are highly variable in terms of both content and profile. The results of the current study for first and follow-on formulas coincide with those of Pollack et al. [8], Buts et al. [9], and Atiya-Ali et al. [12]. However, much higher values have been reported by other authors, with levels reaching 964 nmol/dL, 923 nmol/dL, and 712 nmol/dL for putrescine, spermidine, and spermine, respectively [23]. A study by Gómez-Gallego et al. [22] indicated that the polyamine content in infant formulas depends mainly on the raw milk composition but also on the manufacturing process. They also suggest that the activity of polyamine oxidase, an enzyme found in raw milk, may be responsible for changes in polyamine concentrations, as it seems resistant to the skimming, pasteurization, concentration, and drying processes used in formula production [22]. Thus far, no other study has related the activity of polyamine oxidase with the variability of polyamine content in infant formulas, and further work is required to elucidate if the differences among products are the result of polyamine interconversion reactions as well as enzymatic degradation.

Table 1 also shows the polyamine contents of infant formulas for special medical use prepared from plant proteins (rice and soy), which are a bit more variable compared to conventional cow’s milk formulas, not only among brands but also batches. Significant differences among brands and batches are displayed in Table 1. Rice-based formulas stand out for high putrescine levels, which range from 188 to 312 nmol/dL and are significantly higher than in soy-based and cow’s milk formulas (*p* < 0.05). In contrast, spermidine predominated in soy-based formulas (179–337 nmol/dL) (*p* < 0.05). Spermine was not detected in any plant-based infant formulas, which can be attributed to its lower presence in products of plant origins.

The only prior study on polyamines in soy-based formulas, performed by Romain et al. (1992) [7], obtained very similar results for putrescine (74 nmol/dL) and spermidine (256 nmol/dL) but also found low levels of spermine (44 nmol/dL). No previous data on polyamines in rice-based infant formula are available in the literature.

In plant protein-based infant formulas, the polyamine content and profile are also clearly related to the raw materials, with putrescine and spermidine being predominant in rice and soybean, respectively [35,38]. Thus, the differences in polyamine content among brands and batches can be attributed not only to differences in the manufacturing processes but also to the characteristics of the raw materials (i.e., rice or soybean). The levels of polyamines in plant foods and, therefore, in derived products such as infant formulas, can be influenced by several factors, including cultivation, harvesting, and environmental conditions (e.g., droughts) [25,39,40,41].

**Figure 3:**
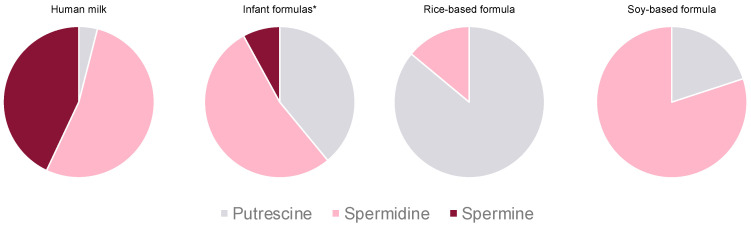
Polyamine distribution in human milk and infant formulas. * First, follow-on and preterm infant formulas.

Regarding the polyamine profiles, a high variation was observed when comparing the samples of human milk and the different types of infant formulas (Figure 3). In conventional infant formulas, the proportion of putrescine was far higher than in human milk (where spermidine and spermine predominated), with spermine being found only in two preterm formulas (hence its low proportion overall). Differences were particularly striking among human milk and plant protein-based formulas, with those based on rice and soy being characterized by a hegemonic predominance of putrescine and spermidine, respectively.

In conclusion, polyamines were detected in all human milk samples during the first five months of lactation, with spermidine and spermine being the predominant compounds. Considerable differences were observed in polyamine content and profile among human milk and infant formulas. In fact, polyamine concentrations were up to 30 times lower in infant formulas, with putrescine being the predominant polyamine. Bearing in mind the importance of polyamines in the early stages of life, the results of the current study indicate that the polyamine content of infant formulas should be improved, both qualitatively and quantitatively, to more closely match the composition of human milk.

## Figures and Tables

**Table 1:** Polyamine content (nmol/dL) in different types of infant formulas.

Infant Formulas	Batch	Putrescine	Spermidine	Spermine
		Mean ± SD	Mean ± SD	Mean ± SD
First formula				
A1	1	Nd	10.7 ± 0.5	Nd
	2	Nd	20.7 ± 1.0	Nd
A2	1	40.3 ± 0.8	26.5 ± 0.5	Nd
	2	33.5 ± 0.8	18.6 ± 2.0	Nd
A3	1	36.3 ± 1.6	45.1 ± 1.5	Nd
	2	37.4 ± 1.6	42.3 ± 1.4	Nd
Follow-on formula				
B1	1	40.3 ± 0.8	32.7 ± 2.4	Nd
	2	42.5 ± 0.8	39.2 ± 1.0	Nd
B2	1	35.7 ± 0.8	32.0 ± 1.5	Nd
	2	34.6 ± 0.8	32.7 ± 0.5	Nd
B3	1	59.6 ± 0.8 **#**	47.2 ± 4.8	Nd
	2	57.3 ± 0.8 **#**	56.8 ± 4.3	Nd
Preterm formula				
C1	1	47.1 ± 0.8	29.3 ± 0.5	62.0 ± 1.1
	2	38.0 ± 0.8	24.8 ± 1.0	61.7 ± 2.8
C2	1	Nd	226.9 ± 3.4 **#**	63.7 ± 7.7
	2	Nd	229.6 ± 7.8 **#**	61.5 ± 3.8
C3	1	39.1 ± 0.8	33.4 ± 0.5	Nd
	2	39.1 ± 0.8	33.4 ± 0.5	Nd
Rice-based formula				
D1	1	188.9 ± 12.0 †	18.6 ± 2.0	Nd
	2	191.7 ± 6.4 †	13.8 ± 2.9	Nd
D2	1	199.7 ± 1.6 †	43.7 ± 3.4	Nd
	2	188.3 ± 4.8 †	52.7 ± 0.5	Nd
D3	1	306.3 ± 28.9 †	53.7 ± 6.8 *	Nd
	2	311.9 ± 24.1 †	19.6 ± 1.5 *	Nd
Soy-based formula				
E1	1	70.3 ± 1.6	337.4 ± 2.9 *†	Nd
	2	77.7 ± 0.8	278.5 ± 0.5 *†	Nd
E2	1	43.1 ± 1.6	179.0 ± 1.0 *†	Nd
	2	60.7 ± 4.0	225.8 ± 1.9 *†	Nd
E3	1	74.9 ± 1.6	303.6 ± 2.9 *†	Nd
	2	70.3 ± 3.2	258.2 ± 4.9 *†	Nd

Nd: not detected. One-way ANOVA employing T3 Dunnett was used to compare the polyamine content in infant formulas. Different symbols indicate statistical significance. # *p* < 0.05 differences among brands in putrescine in B3 follow-on formulas and spermidine in C2 preterm formulas. † *p* < 0.05 differences in putrescine in rice-based formulas and spermidine in soy-based formulas among all infant formulas. The Student *t* test was used to compare the polyamine content between batches. * *p* < 0.05 differences in spermidine in rice-based formulas (D3) and soy-based formulas (E1, E2, E3).
